# Automatic Detection of Freshwater Phytoplankton Specimens in Conventional Microscopy Images

**DOI:** 10.3390/s20226704

**Published:** 2020-11-23

**Authors:** David Rivas-Villar, José Rouco, Manuel G. Penedo, Rafael Carballeira, Jorge Novo

**Affiliations:** 1 Centro de investigación CITIC, Universidade da Coruña, 15071 A Coruña, Spain; jrouco@udc.es (J.R.); mgpenedo@udc.es (M.G.P.); jnovo@udc.es (J.N.); 2 Grupo VARPA, Instituto de Investigación Biomédica de A Coruña (INIBIC), Universidade da Coruña, 15006 A Coruña, Spain; 3 Centro de Investigacións Científicas Avanzadas (CICA), Facultade de Ciencias, Universidade da Coruña, 15071 A Coruña, Spain; r.carballeira@udc.es

**Keywords:** microscope images, phytoplankton detection, colony merging, gabor filters, bag of visual words

## Abstract

Water safety and quality can be compromised by the proliferation of toxin-producing phytoplankton species, requiring continuous monitoring of water sources. This analysis involves the identification and counting of these species which requires broad experience and knowledge. The automatization of these tasks is highly desirable as it would release the experts from tedious work, eliminate subjective factors, and improve repeatability. Thus, in this preliminary work, we propose to advance towards an automatic methodology for phytoplankton analysis in digital images of water samples acquired using regular microscopes. In particular, we propose a novel and fully automatic method to detect and segment the existent phytoplankton specimens in these images using classical computer vision algorithms. The proposed method is able to correctly detect sparse colonies as single phytoplankton candidates, thanks to a novel fusion algorithm, and is able to differentiate phytoplankton specimens from other image objects in the microscope samples (like minerals, bubbles or detritus) using a machine learning based approach that exploits texture and colour features. Our preliminary experiments demonstrate that the proposed method provides satisfactory and accurate results.

## 1. Introduction

In order to asses water safety and quality, it is necessary to perform toxic phytoplankton analysis and quantification. This procedure is usually performed by expert taxonomists by manual counting on the microscope. Expert assessment usually achieves low recall rates (75%) in water samples with high debris concentration [1], and mean species identification rates below 73.7% [2]. Manual phytoplankton analysis also shows lack of agreement between experts and criteria discrepancies of the same expert in different conditions. The variation among experts is reported to be approximately 42% or 43% [3,4]. The disparity in the assessment of the same expert has been measured to be between 67% and 83% [3]. These procedures may be automated to improve the objectivity and repeatability of the process, releasing the experts from tedious and time-consuming work.

The state of the art automatic procedures are focused on several stages of the process: (1) the sample gathering, (2) the automatic detection and segmentation of the specimens, and (3) the identification and counting of species. This work is focused on providing a novel solution to the automatic detection and segmentation of phytoplankton specimens is conventional microscope images, aiming at the application of automatic identification and quantification techniques using readily available equipment in the laboratories.

Data gathering in state-of-the-art solutions usually relies on robotic contraptions that can capture images of plankton specimens in the water without human intervention. Some notable examples are Shadowed Image Particle Profiling and Evaluation Recorder (SIPPER) [5], Video Plankton Recorder (VPR) [6,7], FlowCytobot [8] and KRIP [9] or FlowCam [3,10,11], among others [12,13,14].

On the other hand, the automatic detection and segmentation of phytoplankton specimens is approached in a different way depending on the kind of images acquired from each device. In this regard, some of the previous devices, like VPR [6,7] or KRIP [9], provide images with multiple specimens. In these works, simple image processing approaches have been proposed to detect and segment each separate specimen in the images. However the detection and segmentation methods used are usually designed ad-hoc for the imaging features of each device. Differently, other devices like FlowCytobot [8], FlowCam [3,10,11] and others [5,12,14] use flow cytometry imaging techniques to directly obtain single specimen images during the imaging process. Cytometry works by passing the water sample through a thin tube in which only a single specimen can fit, and performing the microscopic imaging there. While these techniques allow to separate each specimen in a single image, they require to change of the flow cell depending on the size of the specimens to be analysed [15]. Moreover, an additional classification stage is necessary to differentiate between target phytoplankton specimens and other objects in the water sample, which are also detected and imaged.

Finally, the taxonomic identification and counting of the phytoplankton species is usually approached using machine learning approaches applied to single-specimen images [16,17,18,19,20,21,22]. Most of the existent works use images obtained with flow imaging approaches, like FlowCytobot [21,22], FlowCam [16] or other types of cytometer [20,23].

The taxonomic identification from single-specimen images has experimented a remarkable research interest in many recent works, and achieved satisfactory results on several identification targets. In this sense, some works have focused on the phytoplankton identification at the genus level [22,24], while others are focused on the accurate identification of a limited set of target species [20,21]. It is also common to focus on only identifying members of a single group like diatoms [17,18,19].

In these works, several machine learning approaches have been explored, ranging from classical approaches to deep learning. For example, in regards to classical machine learning methods, several works have explored SVMs [24,25] as well as a variety of other methods [2,19,22]. Deep learning has also been tested in this topic, particularly CNNs of various designs [17,20]. Moreover, transfer learning was also successfully applied to taxonomic identification of phytoplankton [21].While there are still some open issues in the taxonomic identification of single-specimen phytoplankton images, these are out of the scope of this paper. Nevertheless, it should be noted that most of the identification advances of the existent approaches, especially the most recent works based on deep learning, can be directly applied to any single-specimen image regardless of its imaging source.

Thus, the access to most of the recent advances in automatic phytoplankton analysis is tied to ability to automatically obtain single-specimen images of phytoplankton. However, the state of the art is dominated by the use of automatic imaging devices which are not readily available in most of laboratories worldwide. Instead, the most prevalent phytoplankton analysis method is to manually gather and process the water samples and manually inspect them using regular light microscopes. In this sense, as regular microscopes equipped with digital cameras are widely available, it is relatively affordable to obtain digital images of water samples from the routine work of laboratory technicians. Thus, it would be desirable to provide automatic analysis solutions that can take advantage of these conventional microscope images, so that an automatic analysis solution, aiming at releasing the expert taxonomists from tedious identification and counting works, can be made available at low cost. However, to the best of our knowledge, these images have not been the target of automated analyses, with the exception of some few related works [26,27,28].

Some of these related works propose to use fluorescence imaging and the integration of images with multiple focal points [26,27], or even multiple magnifications [27]. While this eases the detection and segmentation of phytoplankton specimens, it involves a complicated imaging protocol, which probably requires the use of computerised microscopes [26,27]. In other works [28], the proposed imaging method can not even be performed in a systematic way, as they require an human expert to select the appropriate focal point and magnification used for each of the imaged species. Conversely, this work is focused on the analysis of conventional microscope images that are acquired using a systematic imaging protocol aiming at minimising the required human intervention. Specifically, the images are captured at a fixed magnification and focal point which could allow its application without the intervention of an expert taxonomist.

In this work, we propose a novel fully automatic computational approach to detect and separate the individual phytoplankton specimens that are present on multi-specimen microscope images. The proposed solution aims to be a preliminary approach that aims being able to systematically apply the most recent advances in automatic phytoplankton identification using conventional microscopy. Detecting separate specimens in microscope water samples is a challenging task due to the high variability of the phytoplankton species, specially in fresh water samples, and the presence of diverse types of debris.

The proposed preliminary approach consists in a fully automatic pipeline based on classical algorithms for segmentation and novel methods for the fusion of specimens. This method also makes use of machine learning approaches capable of differentiating phytoplankton from the many spurious objects present in the images like zooplankton, garbage or minerals by exploiting texture and colour information. This preliminary approach is a baseline for further research that intends to continue advancing in this area.

## 2. Methodology

**Figure 1:**
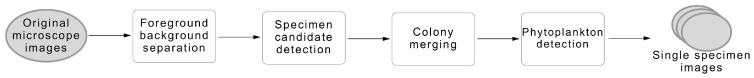
Main steps of the proposed methodology.

The proposed fully automatic methodology for phytoplankton specimen detection, represented in Figure 1, is divided into four different main steps. The foreground-background segmentation stage binarizes the image separating both classes. Then, every candidate region in the foreground-background map is detected, discarding the ones that do not fit some domain-related selection criteria. To improve the results, a novel accurate merging algorithm is proposed to allow the fusion of sparse individuals or colonies into a single detection. The final step aims at distinguishing the real existing phytoplankton specimens from other objects and artefacts in the image using a machine learning-based approach.

### 2.1. Foreground-Background Separation

The first step separates any existing foreground from the water background for the posterior analysis. For this purpose, we consider that the foreground includes a variety of objects apart from phytoplankton like zooplankton, garbage, sand particles, etc. Due to the capture process, the images also have other kind of imperfections, such as shadows or uneven illumination which complicate this step. The proposed foreground-background separation consists in thresholding each of the RGB channels independently, and then combining the results. An adaptive Gaussian threshold [29] is used for this in order to obtain a stable and robust segmentation under the presence of variable noise or uneven illumination, which is common in the analysed images. The adaptive threshold for each image position is computed as the Gaussian-weighted local average of each image channel, minus a fixed value *C*. The Gaussian window spread is set to *σ* = 75 μm which is large enough to ensure that the window is larger than the target cells. The offset is empirically set to C=8% of the dynamic range, to improve the method sensitivity. Once the three channels are thresholded, they are merged using an OR operator to preserve the highest amount of information among the three channels.

**Figure 2:**
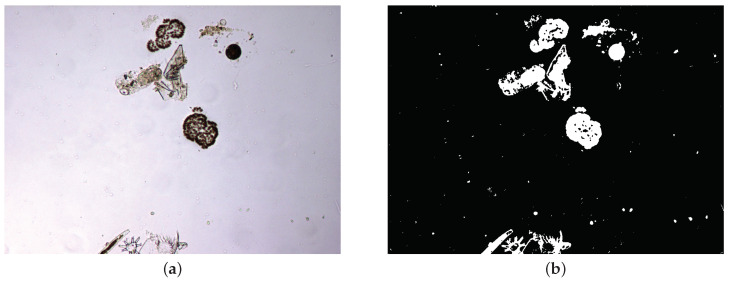
Example of foreground-background separation. (**a**) Original image. (**b**) Binary map separating the foreground from the background.

The output of this step, illustrated in the example of Figure 2, is a binary map that separates foreground and background.

### 2.2. Specimen Candidate Detection

To separate the different existing specimens, a blob detection process is performed using the Suzuki and Abe’s algorithm [30]. This algorithm detects the individual connected regions by tracing their contours. We consider each separated component as a preliminary candidate. The external contour of each region, as provided by the method, is considered as the entire candidate segmentation, i.e., the internal holes are filled in.

Once each preliminary candidate has been detected, they are analysed and candidates with areas below 5 μm^2^ are discarded as being considered not representative and possibly misleading for the analysis. The exclusion of solitary cells < 5 μm is not a general consideration but a criterion that we have adopted due to a limitation of image resolution. Solitary cells with a major axis less than 5 μm are too inconspicuous to establish correct taxonomic identification, even at 40× magnification, by an experienced taxonomist. In this work we consider 10× magnification to increase the imaging field size, as well as enabling the imaging of complete specimens in a single image. Thus the resolution issue is even worse in this case. The used parameter value is derived accordingly, depending on the image resolution. It was decided to use an area criterion as it allows more shape diversity than using the size along an arbitrary axis of the segmented blobs. Additionally, blobs on the edges of the image have been discarded, since we have also established as a routine counting criterion that any partially included specimens located on the edges of the image will not be taken into account [31,32,33]. This prevents the inclusion of partial specimens, which may induce identification and quantification errors in further processing stages. Moreover, this exclusion does not affect the overall quantitative analysis of a water sample, provided that such analysis will include a high number of imaged fields.

### 2.3. Colony Merging

The previous step is able to correctly segment most of the specimens of the analysed images. However, for sparse specimens as well as phytoplankton colonies, which are very common, the phytoplankton specimens are detected as several candidates. The reason for this sparse detection as several candidates is that the physical bonds between the individual cells of the specimens are not visible in the microscope images. However, the joint detection of these candidate as a single specimen is required in order to correctly identify the species in subsequent taxonomic classification processes.

**Figure 3:**
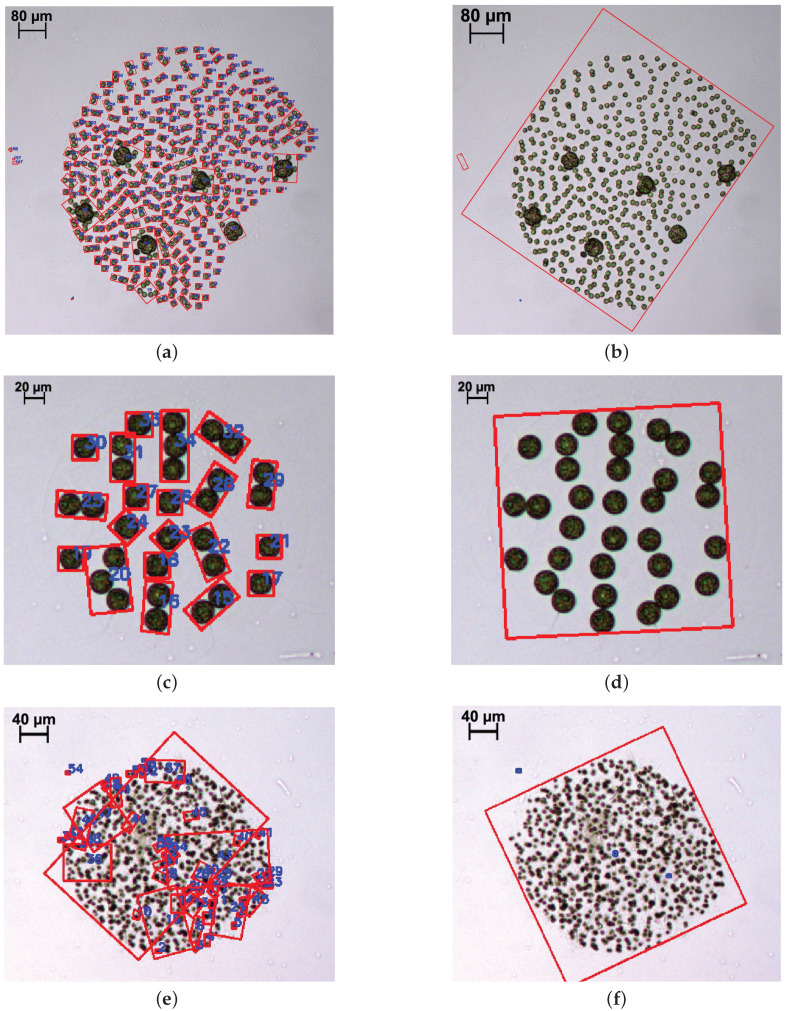
Example of colony merging results over particular specimens (not to scale). (**a**) *Volvox aureus* detected in separated parts. (**b**) Same *Volvox aureus* after the merging algorithm. (**c**) *Eudorina elegans* detected in separated parts. (**d**) Same *Eudorina elegans* after the merging algorithm. (**e**) *Microcystis flos-aquae* detected in separated parts. (**f**) Same *Microcystis flos-aquae* after the merging algorithm.

For that reason, we designed an extra merging stage with a new, robust and precise method to obtain adequate unique identifications of phytoplankton colonies and sparse specimens. Representative examples of this are presented in Figure 3, where the detection results prior to this step (first column) are compared to the results after this proposed merging process (second column).

**Figure 4:**
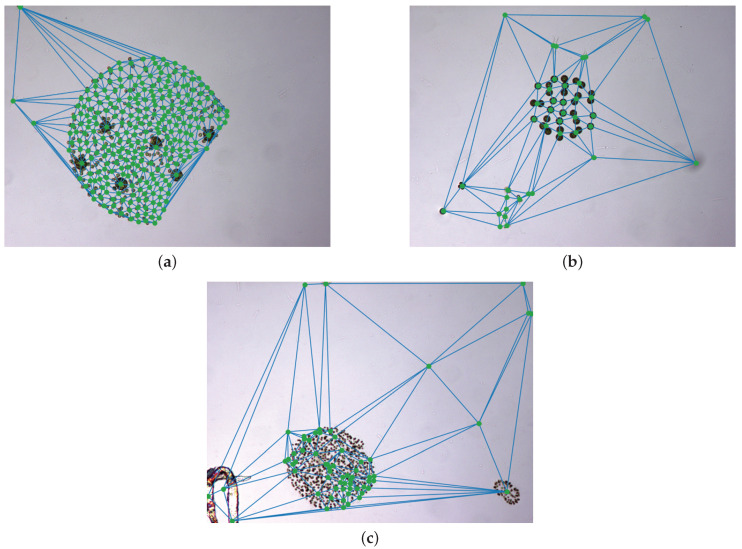
Examples of Delaunay triangulations over images containing different sparse species or colonies. (**a**) *Volvox aureus*. (**b**) *Eudorina elegans*. (**c**) *Microcystis flos-aquae*.

The new proposed method is based on the fusion of nearby candidates that share similar visual features. In order to perform this analysis, we first create a graph containing every detection in an image using a Delaunay triangulation [34,35] of the candidate centroids. This allows to link each detection with its nearby neighbours. Some examples of this triangulation are depicted in Figure 4 over different images where varied species are the target of this fusion. Then, the algorithm compares each pair of connected nodes in the triangulation. The comparison is performed in terms of spatial distance and colour similarity. If the Euclidean distance between the node positions is above a certain threshold, the link is pruned. Likewise, if the distance between the colours of the candidates is above a certain threshold the link is pruned. Specifically, the empirically selected thresholds are 105 μm in distance combined with a difference of 15% of dynamic range per RGB channel. The colour value for each candidate is computed using the average RGB value of the pixels inside its segmentation mask. The use of the segmentation mask, instead of the whole bounding box, prevents the background skewing the estimated colour. Once all the node pairs have been explored, the remaining connected subgraphs are fused into single candidates. As previously mentioned, the results of this whole process can be observed and compared to the preliminary detections in Figure 3, for which Delaunay triangulations are shown in Figure 4, respectively. In particular, these images show three different examples of fusions applied to a varied set of species: *Volvox aureus*, *Eudorina elegans* and *Microcystis flos-aquae*. These specimens are not equally oversegmented in the first step and, therefore, present different challenges for the fusion algorithm.

### 2.4. Phytoplankton Detection

This final step aims at classifying candidate specimens into phytoplankton specimens and other objects that are present in the image, like organic garbage, minerals or zooplankton, among others. To carry out this objective, the previous detections are used to train a classifier that differentiates both classes, filtering therefore undesirable detections from the target ones.

This step uses the previously detected specimens with their bounding box grown a 10% more in each direction to include a better context with some background information.

To classify this data, we have explored the use of several alternative classifiers using colour and/or texture features. In particular, the tested classifiers are Random Forest (RF) [36], Support Vector Machines (SVM) [37], k-Nearest Neighbours (kNN) [38], Boosting Trees (BT) [39] and Gaussian Mixture Models (GMM) [40]. Besides, the considered features are obtained using a Bag of Visual Words (BoVW) [41,42] either using RGB values or Gabor filter banks [43] to represent colour or texture respectively. Both colour and texture features are characteristic of the phytoplankton species thus we experimented using each of them independently or both together. We have not considered the use of explicit shape information due to the high number of possible species and of debris in fresh water samples. Instead, the objective is to capture global common appearance of phytoplankton to differentiate it from other non-target objects in the images.

The BoVW model allows to represent an image region through histograms of visual patterns (words). The visual words consist in local patterns characterised by specific combinations of base visual features. The specific combinations defining the visual words are learned using a clustering algorithm and stored into a dictionary posteriorly used as reference to compute the histograms. In this work, K-means clustering [44] is used to create the dictionary of visual words for either colour or texture features. Generally, the base visual features for each of these cases are determined below.

#### 2.4.1. Colour Features

The colour features are obtained using the RGB values for each image position as base visual features of the BoVW model. All the bounding boxes detected using the previous stages in the training set are used to compute the colour dictionary. The RGB values inside the bounding boxes are gathered and clustered using *k*-means. A specific number of *k*-means cluster centres $k_{c}$, which is empirically selected during the experiments, is used. The resulting dictionary is used to compute the BoVW histogram that, in this case, represents the distribution of colours within the bounding box. It should be noted that this colour representation also provides some implicit shape features, as the background colours are also included in the bounding boxes and thus the BoVW histograms would also represent the background-foreground proportions within the bounding box.

#### 2.4.2. Texture Features

In this case, the set of complex responses to a Gabor filter bank act as base features for each image position in the BoVW model. Thus, the visual words represent local shape patterns, and the histograms of these patterns along the whole regions represent texture.

The used filter bank consists of Gabor filters of a single scale (central frequency) and bandwidth, and a varying number of orientations. The complex Gabor filter for each orientation θ is given by

(1) $$\begin{array}{c} g\left(x,y;\theta \right)=\frac{f_{c}^{2}}{\pi \sigma^{2}}e^{f_{c}\left(\frac{x^{\prime 2}y^{\prime 2}}{\sigma^{2}}\right)}e^{-j2\pi f_{c}x^{\prime }}, \end{array}$$

where $f_{c}$ denotes the central frequency of the filters, *j* denotes the imaginary part and σ controls the spread of the isotropic envelope. The $x^{\prime }$ and $y^{\prime }$ coordinates are a rotated space according to

(2) $$\begin{array}{c} x^{\prime }=x\cos\theta +y\sin\theta ,\\ y^{\prime }=-x\sin\theta +y\cos\theta , \end{array}$$

where θ denotes the rotation angle. The σ value is computed depending on the central frequency $f_{c}$ according to

(3) $$\begin{array}{c} \sigma =\frac{1}{\pi f_{c}}\cdot \sqrt{\frac{\ln2}{2}}\cdot \frac{2^{B}+1}{2^{B}-1} \end{array}$$

where *B* denotes the filters bandwidth in octaves.

The bank of filters is composed of $N_{o}$ filters with $\theta \in \{\theta ,\pi /N_{o},\cdots ,\pi \left(N_{o}-1\right)/N_{o})\}$ corresponding to $N_{o}$ evenly distributed orientations. As we consider both real and imaginary filters this results in a total of $2N_{o}$ responses for each image position.

The central frequency $f_{c}$, the frequency bandwidth *B*, the number of orientations $N_{o}$ and the number of *k*-means clusters $k_{t}$ are left as free parameters and their values are selected empirically during the experiments.

The filter bank responses are processed in a similar way to colour features, by considering all the pixels within the bounding boxes. However, in this case, the filter bank responses are computed over the whole image, prior to splitting it into bounding boxes, to minimize the aliasing effects due to the image boundaries. Also, the input image is converted to grayscale for the texture description procedure.

## 3. Experimental Setup

### 3.1. Phytoplankton Sampling and Microscopy

A 2 L water sample was collected at a depth of 3 m using a van Dorn bottle in the coastal freshwater lake of Doniños (Ferrol, Galicia, Spain) (UTM 555593 X, 4815672 Y; Datum ETRS89) on 16 November 2017. The phytoplankton sample was concentrated by filtering volume of 0.5 L through GF/F glass fiber filters and was then resuspended in 50 mL. Phytoplankton samples were preserved using 5% (*v*/*v*) glutaraldehyde, because it is efficient at preserving both cellular structures and pigment [45,46,47,48]. The fixed sample was stored in the dark at constant temperature (10 °C) until analysis.

The phytoplankton sample was homogenised for 2 min prior to microscopic examination. In addition, the sample was subjected to vacuum for one minute to break the vacuoles of some cyanobacterial taxa and prevent them from floating. The data on the taxonomic composition and abundances of the different taxa in the water sample, characterised by quantitative analysis in a Ütermohl sedimentation chamber [49] using a Nikon TMD inverted microscope equipped with a Plan Phase Contrast 40× objective (N.A. 0.60), is detailed in Table S1. In summary, the sample had a density of 9018 cells per ml, with a total of 51 taxa and the following relative abundances of the main taxonomic groups: Chlorophyceae 56.77% (i.a. *Botryococcus braunii* Kützing, *Volvox aureus* Ehrenberg, *Dictyosphaerium pulchellum* H.C. Wood), Cyanobacteria 24.95% (i.a. *Woronichinia naegeliana* (Unger) Elenkin, *Microcystis flos-aquae* (Wittrock) Kirchner, *Anabaena spiroides* Klebahn) and Bacillariophyceae (diatoms) 15.29% (i.a. *Aulacoseira granulata* var. *armata* (Ehrenberg) Simonsen, *Fragilaria crotonensis* Kitton).

### 3.2. Digital Image Dataset

Aliquots of the phytoplankton sample with a total volume of 1 mL were examined under light microscopy using a Nikon Eclipse E600 equipped with an E-Plan 10× objective (N.A. 0.25). Light microscopy images were taken with an AxioCam ICc5 Zeiss digital camera (12 Megapixels), maintaining the same illumination and focus throughout the image acquisition process and following regular transects until the entire surface of the sample was covered.

However, taking images at this magnification complicates the systematic application of an automatic approach due to several reasons. First, because there is size heterogeneity and diversity of taxa inherent to phytoplankton samples, which prevents the observation of whole organisms of some species at 40× magnification. This also means that careful ad-hoc framing of the imaging field by the microscope operator for most specimens would be required. Second, the aperture of large magnification objectives is usually larger, resulting in a much lower depth of focused plane. Thus, using 40× magnification would usually require varying the focus while examining each individual. Finally, the larger magnification also means a smaller field of view. Therefore, the use of 40× objectives would require the imaging of a larger number of transects to cover the whole sample field. Reducing the magnification to 10× minimises all these drawbacks and provides more suitable images for the automatic analysis.

The resulting digital image dataset is comprised of 211 digital microscopy images obtained from the described water sample. The 12 Mpx at 10× magnification result in an approximate resolution of 1.5 pixels per μm. Each of these images contain several phytoplankton specimens of varying taxa. Overall, the set of images contains 1209 true phytoplankton specimens that can be divided among 51 different species along with a significant amount of other objects like zooplankton, inert organic matter, minerals, etc, which are identified as non-phytoplankton.

A random subset of 50 images was used for training while the rest were reserved for testing. The ground truth consists of bounding boxes enclosing the phytoplankton specimens, which were marked by an expert.

### 3.3. Experimental Evaluation

The experimentation consisted in the evaluation of the initial candidate detection, quality of segmentation and the final phytoplankton detection.

The candidate detection stage is evaluated in terms of false negative rates (FNR). Detections are counted as positive when their bounding box overlaps at least 50% of the true specimen. The parameters of the foreground-background separation and the initial candidate detection stage are empirically optimised to minimize the FNR by only observing the training set.

The quality of the segmentation is evaluated by the production of over and undersegmentations. Oversegmentation occurs when two or more boxes cover a specimen that should only be detected by one. Undersegmentation is the opposite, two or more specimens are enclosed in the same bounding box. This evaluation also serves to measure the impact of the novel colony merging stage. To that end, the parameters of the colony merging are selected to provide a satisfactory under/over-segmentation balance in the training set.

The final phytoplankton detection is evaluated using precision at high levels of recall, namely 90% and 95%. This is due to the intention of preserving most of the phytoplankton specimens in an imbalanced dataset. Additionally, precision-recall (PR) curves are used to illustrate the performance of the optimised systems. PR curves are also used instead of Receiver Operating Characteristic (ROC) curves due to the imbalanced nature of the problem.

For the phytoplankton detection stage, we tested RF, SVM, kNN, BT and GMM classifiers with the described colour and texture feature sets alone or both. For each of these classifiers, using either colour or texture features (i.e., 5×2 alternatives), we performed a cross-validated grid search over the training set, to select the optimal parameters. To that end, Precision at 90% recall was used as the fitness measurement for the parameter selection. This metric was chosen instead of the more common Area Under Curve (AUC) for the PR curves due to cases where the AUC was misleading, reporting high values for systems with poor precision at high levels of recall. This meant that, even if the overall AUC was high due to the system performing well under lower quantities of recall, it could not be accepted due to the poor performance at the highest levels. As in this work we are focused on detecting the highest amount of true phytoplankton, therefore, precision at 90% recall more akin to our goal.

The grid search included hyper-parameters of the classifiers along with the free parameters of the proposed BoVW descriptors. Specifically, the number of visual words ($k_{c}$ and $k_{t}$, for colour and texture, respectively) was selected among 100, 50, 20, 10, 8, 5, 3 and 2. Additionally, for the texture descriptors, the centre frequency $f_{c}$ is selected among 0.5, 0.3535, 0.25, 0.177, 0.125, 0.088, 0.0625, 0.0442, 0.03125, 0.0221, 0.0156 and 0.01105 $px^{-1}$; the bandwidth B among 2, 1.5, 1 and 0.5 octaves; and the number of orientations $N_{o}$ between 4 and 8. Once the best descriptor configurations are found, the BoVW dictionary is computed for each alternative using the whole training set. These BoVW dictionaries are kept fixed to compute the descriptors over the test set.

Finally, a second cross-validated grid search is performed again over the training set to optimize the classifier hyper-parameters with the optimal descriptors. In this case, we consider three descriptor options for each classifier (colour, texture or both concatenated). The results of these 5×3 classification alternatives are reported to provide a comparison.

The comparison with other methods in the state of the art was not considered in this work due to the novelty of this approach and the relative lack of works treating this issue. This is especially notable when looking at the modality of images we employ since, as far as we know, no other work neither automatic nor semiautomatic makes use of regular microscopy images despite them being common and relatively affordable to obtain. Moreover, the BoVW model allows to integrate feature extraction into the machine learning pipeline, using base features of diverse information sources, as colour or texture. The exhaustive comparison with competing alternative methodologies to represent colour and texture is out of the scope of this preliminary work.

## 4. Results and Discussion

The specimen candidate detection step results in a FNR of 0.4% using the test set, which is equivalent to 4 missed specimens among the total of 994 that are present in the test set. The low FNR is motivated by the fact this initial stage aims at identifying as much phytoplankton specimens as possible. Consequently, 851 incorrect detections were also provided, detections that are posteriorly filtered in the corresponding stage. These false positives are zooplankton and inert organic matter that present similar appearances to our phytoplankton. They are visually difficult to classify due to the intra-class heterogeneity of phytoplankton species. In spite of that, phytoplankton shows some distinctive features when compared to the spurious objects in these images. This is why a learning-based approach was used for the candidate refinement stage.

Sparse specimen and colony merging is evaluated by the comparison of the over and undersegmentation metrics before and after the application of this step. In particular, before the colony merging step, we obtained ratios of 20.36% and 8.86% for over and undersegmentation, respectively. Thanks to the application of the merging process, oversegmentation was reduced to a 3.43% while undersegmentation increased to a 12.53%. Thus, the fusion algorithm presents a positive impact given it improves oversegmentation ratio approximately a 17% while only increasing the undersegmentation approximately a 4%. These metrics show that the dataset presents a non-negligible amount of overlapping between specimens, responsible for the original measurement of undersegmentation. The metrics also demonstrate the abundance of sparse specimens and colonies characterised by their transparency and lack a visual connectivity, making them hard to detect as a whole object in the previously stage. These results are satisfactory as some undersegmentation increase is bound to happen when merging specimens, however, it is much lower, than the decrease in oversegmentation. Therefore, we can conclude that the algorithm satisfactorily balances the merging of the oversegmented specimens without resulting in too many false fusions.

**Figure 5:**
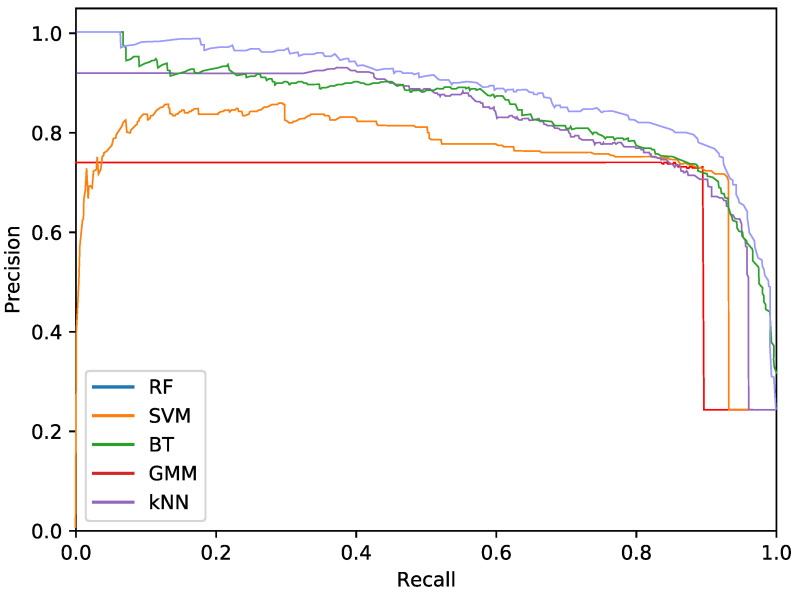
Precision-recall curve for the best performing classifiers using only texture features.

**Figure 6:**
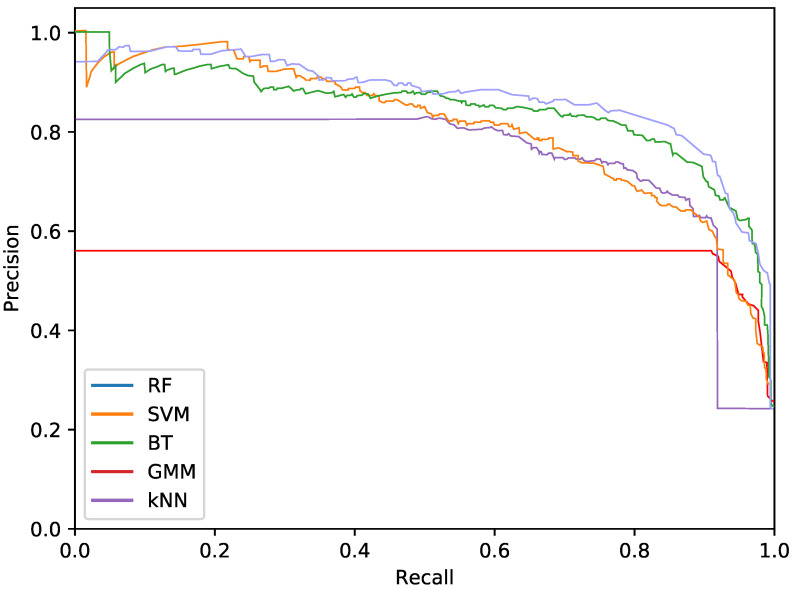
Precision-recall curve for the best performing classifiers using only colour features.

**Figure 7:**
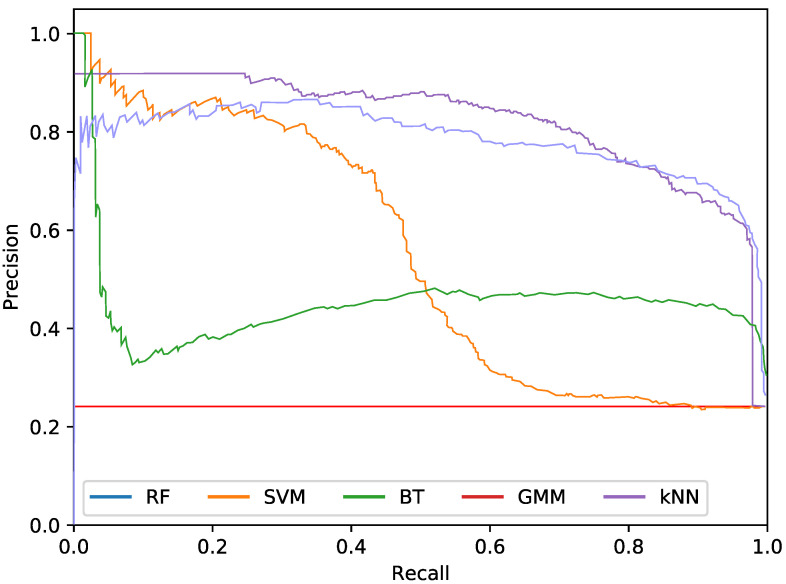
Precision-recall curve for the best performing classifiers using both colour and texture features.

Regarding the phytoplankton detection, Table 1, Table 2 and Table 3 show the performance of the tested classifiers with the different feature sets: texture or colour alone or both together. Complementarily, Figure 5, Figure 6 and Figure 7 show the PR curves for the different classifier and feature set combinations.

As it can be observed in the tables and figures, the performance of several combinations of classifiers and feature sets are similar, therefore validating the suitability of our proposed pipeline. We consider these results satisfactory given the high complexity, the heterogeneous classes and the high variability of the specimens that the system is trying to identify (the large diversity of appearance of the different phytoplankton specimens combined with the large variability of other possible present artefacts as sand, garbage, etc.). Furthermore, as demonstrated by the exhaustive experiments, the system is capable of extracting different features that provide reasonable results across a wide variety of representative classifiers.

The best classifier considering precision at 90% of recall is the RF classifier using texture features. In particular, this model obtained a precision of 77.2%. The empirically selected parameters are: number of bins $k_{t}=100$, central frequency $f_{c}=0.25\;{\mathrm{px}}^{-1}$, bandwidth B=1.5, and 8 orientations. Conversely, considering precision at 95% recall, the best system is the SVM using texture features. This model results in a precision of 68.7%. In this case, the selected parameters were $k_{t}=3$, central frequency $f_{c}=0.0312$, bandwidth B=1.5 octaves, and 4 orientations.

The most important difference among the studied classifiers occurs when switching between the input features. Texture features demonstrate the best performances regardless of the classifier. It should be noted that the difference between using only texture or using only colour features is not very relevant for the RF and BT classifiers, which can be quantified as less than a 2% decrease in performance. On the other hand, the rest of the methods present a much higher reduction in performance, when comparing texture with colour features.Remarkably, the GMM approach loses 13% precision at 90% recall when switching from texture to colour features.

In terms of the descriptor size, kNN, BT and RF are favoured using larger configurations (usually k=100), while SVM and GMM achieve better performances using a lower amount of bins, like 2 or 3. This can also be seen in the amount of orientations, as SVM and GMM tend to make use of 4 instead of 8. This explains the much lower precision that is achieved by these methods using colour and texture features together, as they are more heavily penalised by the larger size of the combined descriptor. Differently, kNN, BT and RF are more suitable for a larger number of features.

While using either colour or texture features show appropriate results, using both together does not provide improvement or even degrades the results. Some classifiers (specially RF) maintained acceptable ratios due to the preference for larger descriptors. SVM and GMM, which favour the opposite, decreased in precision. Nevertheless, the fusion of features has proved useful for slightly improving the RF precision at 95% recall.

**Figure 8:**
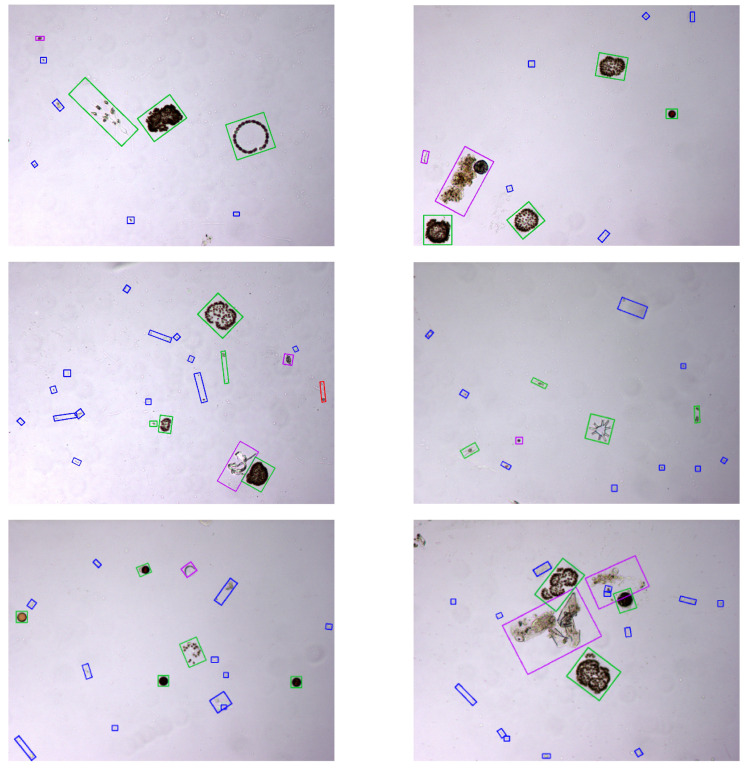
Final results of the work—true positives in green, true negatives in blue, false positives in violet and false negatives in red.

Representative examples of final results of the proposed method are depicted in Figure 8. In particular, several images from the test set are shown with the specimens they contain classified using the top performing system at 90% recall, i.e., RF with the optimal texture features.

By analysing the phytoplankton detection results of the best performing system in the test set, we consider that the proposed approach is satisfactory in differentiating phytoplankton from debris and other objects in the water sample. Specifically, it is remarkable that the number of detected phytoplankton specimens is approximately only half of the detected candidates. Thus, we can conclude that the classification system is robust to the presence of heterogeneous non-target objects in the image. However, these results do not allow to evaluate how robust is the proposed candidate detection mechanism to a very high density of debris and spurious elements in the images. Nevertheless, it should be noticed that the sample concentration can be easily controlled during the water sample preparation through dilution (and the quantitative results corrected consequently) to avoid the exceptionally corrupted samples representing these situations.

One possible limitation of the presented experiments is that the system was tested with images obtained from a single water sample. This implies that the quantity and diversity of non-target objects, as well as the distribution of phytoplankton species and stage of development is biased towards some specific conditions. Nevertheless, we consider that the number of different images is high enough, as well as there is a high number and diversity of both phytoplankton species and non-target objects in the sample to be representative of a realistic scenario. The obtained results, although preliminary, allows us to validate the suitability of the proposed methods, which are to be refined by future research in the same line of work.

Moreover, the present study does not include taxonomic classification and quantification of individual species. Thus, it is not a complete system for phytoplankton analysis. However, such taxonomic classification has been already extensively explored, and successfully approached, in the literature, using the single-specimen images obtained with flow cytometry imaging approaches. The present work allows to obtain single-specimen images from conventional microscopy images that should be readily usable for species classification approaches.

Finally, we have not included a comparison with previous approaches in this work. The reason is that the most related works [26,27,28] do not approach the imaging protocol in a similar way to ours, and thus they are not comparable. Specifically, PlanktoVision [26] and PLASA [27] use fluorescence images along with multiple-focus images obtained using computerized microscopes. Additionally, PLASA also used several magnifications in the analysis. On the other hand, in in [28] the focus and magnification is manually adjusted for each of the imaged species. Thus, in this sense, unlike previous approaches, our method allows to detect single-specimen phytoplankton images from single-focus and magnification images that are systematically obtained using conventional a microscope. Moreover, the comparison with prior species classification approaches is not performed as this classification is out of the scope of this work.

## 5. Conclusions and Future Work

In this work, we present an innovative way of detecting and separating phytoplankton specimens in multi-specimen microscope images from water samples. The designed preliminary fully automatic methodology is based on an initial detection of candidates based on a foreground-background segmentation and a completely novel colony merging algorithm. Finally, given the complexity of this issue, the detections are refined using a learning-based strategy that aims at detecting true phytoplankton specimens. Several classifiers and a wide variety of feature sets were tested, providing in all the cases satisfactory identifications that validate and corroborate the suitability of the proposed automatic computational pipeline.

The results are satisfactory as baseline for further improvement, given the high complexity of the problem and the domain as well as the lack of comparable methods in the state of the art. While the proposed methods need to be tested over more diverse water samples, they can serve as a stable base for future research. In this sense, these are important initial steps towards automating potability testing and plankton studies using conventional microscopes, which are widely available on laboratories worldwide. Overall, the main advantage of this work when compared to other works in this field is its cost and availability. While flow cytometry imaging approaches are suitable methods for obtaining single-specimen phytoplankton images, they are not often found in laboratories. Meanwhile, the proposed methods in this work can be used with a regular microscopes as long as they are equipped with a digital camera. Moreover, and unlike some previous works, since we have managed to use fixed parameters for both magnification and focal point in the imaging protocol, it is not required that an expert taxonomist supervises the image acquisition process. Finally, although species classification and quantification is not approached in this work, state of the art approaches for single-specimen phytoplankton images classification should be easily adapted to work on top of the proposed analysis pipeline.

Future lines of work include testing the method with more phytoplankton samples to verify its performance with a wider amount of species and images. We intend on creating a new classification step to separate relevant species, with a special focus on those that produce toxins. This is the ultimate goal of this project as toxic phytoplankton monitoring has to be done regularly to ensure water safety. We plan on creating a classifier following the same methodology used in this work to classify the toxic species. Finally, other working lines include the exploration of alternative features or deep learning methods.

## Figures and Tables

**Table 1:** Best Result for Each of the Tested Classifiers with Texture Features.

	Precision at 90% Recall	Precision at 95% Recall
RF	77.2%	65.4%
SVM	72.5%	68.7%
kNN	70.4%	61.6%
GMM	69.5%	58.1%
BT	71.7%	59.9%

**Table 2:** Best Result for Each of the Tested Classifiers with Colour Features.

	Precision at 90% Recall	Precision at 95% Recall
RF	75.4%	60.5%
SVM	61.9%	46.3%
kNN	62.6%	46.4%
GMM	56.5%	47.1%
BT	70.9%	62.0%

**Table 3:** Best Result for Each of the Tested Classifiers with Both Colour and Texture Features Combined.

	Precision at 90% Recall	Precision at 95% Recall
RF	69.5%	65.9%
SVM	24.2%	23.9%
kNN	69.3%	49.1%
GMM	31.8%	28.1%
BT	42.9%	44.8%

## Supplementary Materials

The following are available online at https://www.mdpi.com/1424-8220/20/22/6704/s1, Table S1: Detailed cell density and composition of the used plankton sample.

## Abbreviations

The following abbreviations are used in this manuscript:

RGB	Red, Green, Blue
RF	Random Forest
SVM	Support Vector Machines
kNN	k-Nearest Neighbours
BT	Boosting Trees
GMM	Gaussian Mixture Models
CNN	Convolutional Neural Network
BoVW	Bag of Visual Words
FNR	False Negative Rate
PR	Precision-Recall
ROC	Receiver Operating Characteristic
AUC	Area Under Curve
